# A Qualitative Transcriptional Signature for Predicting Extreme Resistance of ER-Negative Breast Cancer to Paclitaxel, Doxorubicin, and Cyclophosphamide Neoadjuvant Chemotherapy

**DOI:** 10.3389/fmolb.2020.00034

**Published:** 2020-03-25

**Authors:** Yanhua Chen, Hao Cai, Wannan Chen, Qingzhou Guan, Jun He, Zheng Guo, Jing Li

**Affiliations:** ^1^Fujian Key Laboratory of Medical Bioinformatics, Department of Bioinformatics, The School of Basic Medical Sciences, Fujian Medical University, Fuzhou, China; ^2^Key Laboratory of Gastrointestinal Cancer (Fujian Medical University), Ministry of Education, School of Basic Medical Sciences, Fujian Medical University, Fuzhou, China; ^3^Medical Big Data and Bioinformatics Research Center, First Affiliated Hospital of Gannan Medical University, Ganzhou, China; ^4^Fujian Key Laboratory of Tumor Microbiology, Department of Medical Microbiology, Fujian Medical University, Fuzhou, China; ^5^Henan Key Laboratory of Chinese Medicine for Respiratory Disease, Henan University of Chinese Medicine, Zhengzhou, China; ^6^Co-construction Collaborative Innovation Center for Chinese Medicine and Respiratory Diseases by Henan & Education Ministry of P.R. China, Henan University of Chinese Medicine, Zhengzhou, China; ^7^Academy of Sciences of Chinese Medicine, Henan University of Chinese Medicine, Zhengzhou, China

**Keywords:** breast cancer, neoadjuvant chemotherapy, pathological complete response, extreme resistance, relative expression ordering

## Abstract

For estrogen receptor (ER)-negative breast cancer patients, paclitaxel (P), doxorubicin (A) and cyclophosphamide (C) neoadjuvant chemotherapy (NAC) is the standard therapeutic regimen. Pathologic complete response (pCR) and residual disease (RD) are common surrogate measures of chemosensitivity. After NAC, most patients still have RD; of these, some partially respond to NAC, whereas others show extreme resistance and cannot benefit from NAC but only suffer complications resulting from drug toxicity. Here we developed a qualitative transcriptional signature, based on the within-sample relative expression ordering (REO) of gene pairs, to identify extremely resistant samples to PAC NAC. Using gene expression data for ER-negative breast cancer patients including 113 pCR samples and 137 RD samples from four datasets, we selected 61 gene pairs with reversal REO patterns between the two groups as the resistance signature, denoted as NR61. Samples with more than 37 signature gene pairs that had the same REO patterns within the extremely resistant group were defined as having extreme resistance; otherwise, they were considered responders. In the GSE25055 and GSE25065 dataset, the NR61 signature could correctly identify 44 (97.8%) of the 45 pCR samples and 22 (95.7%) of the 23 pCR samples as responder samples, respectively; it also identified 13 (16.9%) of 77 RD samples and 8 (21.1%) of 38 RD samples as extremely resistant samples, respectively. Survival analysis showed that the distant relapse-free survival (DRFS) time of the 14 extremely resistant cases was significantly shorter than that of the 108 responders (*P* < 0.01; HR = 3.84; 95% CI = 1.91–7.70) in GSE25055. Similar results were obtained in GSE25065. Moreover, in the integrated data of the two datasets with 94 responders and 21 extremely resistant samples identified from RD patients, the former had significantly longer DRFS than the latter (*P* < 0.01; HR = 2.22; 95% CI = 1.26–3.90). In summary, our signature could effectively identify patients who completely respond to PAC NAC, as well as cases of extreme resistance, which can assist decision-making on the clinical therapy for these patients.

## Introduction

Breast cancer is a common malignancy with the highest incidence and mortality among females (Ferlay et al., 2015; Jia et al., 2015). A standard regimen for estrogen receptor (ER)-negative breast cancer patients, accounting for 30% of breast cancer patients, is paclitaxel (P), doxorubicin (A), and cyclophosphamide (C) neoadjuvant chemotherapy (NAC) (Jemal et al., 2011). However, the heterogeneity of breast cancer can result in different responses to standard therapy (Rouzier et al., 2005; Carey et al., 2007).

In clinical practice, a pathologic complete response (pCR) is defined as a non-viable invasive cancer in the breast and lymph nodes after the completion of NAC, indicating a complete response to NAC and a favorable outcome (Kaufmann, 2003; Guarneri, 2006; Mieog et al., 2007; Liedtke et al., 2008; Rastogi et al., 2008). However, the proportion of pCR is quite low among patients accepting NAC, and most patients have residual disease (RD) (Popovici et al., 2010). Among patients with RD, accounting for a great proportion of patients treated with NAC, most are partial responders, whereas the others are extremely resistant to NAC. These extremely resistant patients cannot benefit from NAC, but only suffer complications resulting from the toxic effects of NAC. More seriously, these patients may lose the best treatment time because clinicians would evaluate the feasibility of curative or conservative surgery after finishing chemotherapy and a series of examinations (Helene et al., 2012). Therefore, the development of a predictor to identify extremely resistant patients who cannot benefit from NAC is of great significance.

Up to now, many signatures have been developed for pCR prediction (Hess et al., 2006; Thuerigen, 2006; Liedtke et al., 2009), but few studies have focused on the identification of extremely resistant patients. The pCR predictive signatures are based on risk scores summarized from quantitative transcriptional data, which have poor reproducibility (Borst and Wessels, 2010; Tabchy et al., 2010; Zhang et al., 2013; Qi et al., 2016) due to widespread batch effects and the uncertain quality of clinical samples. Although several reported quantitative transcriptional disease signatures – including AlloMap^®^ (Pham et al., 2010) – have been approved by the Food and Drug Administration, the tissue samples must be sent to specific laboratories for measurement with strict quality control, which limits their wider applications in clinical practice.

In contrast, qualitative transcriptional signatures based on within-sample relative expression orderings (REOs) are found to be robust against experimental batch effects and can be directly applied to samples at the individualized level (Eddy et al., 2010; Wang et al., 2013; Chen et al., 2017). REO is a binary variable based on comparing the mRNA levels within a single pair of genes (Geman et al., 2004). For a gene pair (*i, j*), the REO pattern represents whether the expression level of *i* is higher or lower than that of *j* in the sample. Additionally, REO-based signatures are also highly robust against common factors that lead to the failure of quantitative transcriptional signatures in clinical applications, such as varied proportions of tumor epithelial cells (Cheng et al., 2017), amplification bias for minimum specimens (Liu et al., 2017), and partial RNA degradation (Freidin et al., 2012; Chen et al., 2017). Thus, the REO-based method is more practicable for tissue biopsy samples acquired by fine needle aspiration (FNA) or core biopsy (CBX) prior to NAC.

Based on the within-sample REOs of gene pairs, Zhang et al. (2013) have developed a pCR predictor and a prognosis predictor for RD to identify patients who might benefit from NAC. However, this study did not consider the impact of ER subtype. ER-positive patients with good prognosis have a lower pCR rate than that of ER-negative patients with poor prognosis (Guarneri, 2006). Meanwhile, for the same set of breast cancer patients approximately 20% of ER states determined by immunohistochemical (IHC) methods gave different results for different pathologists (Dubowitz, 1991; Arihiro et al., 2007), especially for weak ER-positive samples (Hammond et al., 2010; Sheffield et al., 2016), which may reduce the accuracy of pCR prediction. Thus, we re-determined the ER status of breast cancer patients using the 112-gene-pair signature for ER status developed by Cai et al. (2018) to reduce misjudgments of ER status by IHC.

In this study, we used the gene expression data of ER-negative samples reclassified by the 112-gene-pair signature to identify a qualitative transcriptional signature consisting of 61 gene pairs to predict patients with extreme resistance to PAC chemotherapy. Our signature was well-verified in two independent datasets with survival information.

## Materials and Methods

### Data and Preprocessing

We collected four expression datasets (GSE20194, GSE20271, GSE41998, and MDA133) including 250 IHC-determined ER-negative breast cancer patients in total, who accepted PAC NAC, from the Gene Expression Omnibus (GEO^1^) and the MD Anderson Cancer Center^2^ databases. In the datasets of GSE20194 and GSE20271, we only used the expression data of patients who received paclitaxel followed by fluorouracil (F), doxorubicin [or epirubicin (E)], and cyclophosphamide. In the GSE41998 and MDA133 datasets, the treatment regimens for these patients were PFAC and PAC, respectively.

Two other independent expression datasets (GSE25055 and GSE25065) were used to evaluate whether there was a difference in survival between the responsive and the resistant groups. The treatment regimen for patients in GSE25055 was PAC or PA and the treatment regimen for patients in GSE25065 was PA.

Although PAC NAC is a very common chemotherapeutic regimen, doctors design individual drug delivery schemes for each patient, depending on their condition. Some patients received 6 months of NAC including PFAC (e.g., GSE20194), whereas others received sequential NAC starting with 4 cycles of AC administered every 3 weeks, followed by paclitaxel weekly for 12 weeks (e.g., GSE41998). In this study, we only considered the drug type and not the dose of each drug or the duration of chemotherapy. The clinical characteristics for each dataset are summarized in Table 1.

**TABLE 1:** Description of all datasets collected in this study.

Usage	Dataset	Regimen	ER-negative sample size	pCR	RD	With DRFS information
Training	GSE20194 Popovici et al. (2010)	T-FA(E)C^a^	114	46	68	no
	GSE20271 Tabchy et al. (2010)	T-FA(E)C	79	19	60	no
	MDA133 Hess et al. (2006)	T-FAC	51	27	24	no
	GSE41998 Horak et al. (2013)	T-AC^b^	48	29	19^d^	no
Validation	GSE25055 Hatzis et al. (2011)	T-AC;TA^c^	129	45	84	yes
	GSE25065 Hatzis et al. (2011)	TA	68	23	45	yes

*^a^T-FA(E)C paclitaxel (T) followed by fluorouracil (F), doxorubicin (A) [or epirubicin (E)] and cyclophosphamide (C). ^b^T-AC doxorubicin (A) and cyclophosphamide (C) followed by paclitaxel (T). ^c^TA taxane (T) and anthracycline (A) based regimens. ^d^PD and SD samples representing tumor residuals screened from RD samples.*

For the Affymetrix array data, the raw intensity files (.cel), downloaded from the GEO database were processed using the Robust Multichip Average algorithm (RMA) for background adjustment without quantile normalization. The probe identity documents (ID) were mapped to the Entrez gene ID according to the corresponding platform annotation files. If a probe did not map to a gene or was mapped to multiple genes, the data for this probe were deleted. If multiple probes mapped to the same gene, the arithmetic mean of the expression values for the multiple probes was taken as the final expression value for this gene.

### ER Status Re-determination

We used the 112-gene-pair signature developed by Cai et al. (2018) to reclassify the ER-negative samples. An IHC-determined ER-negative patient was reclassified as ER-negative if more than 68 gene pairs match the REOs of the ER-negative signature.

### Identification of the REO-Based Resistant Signature

For each RD (or pCR) sample, the gene expression profile was first converted into a rank profile according to measured expression levels in ascending order (the lowest expression value corresponds to the smallest rank). Then, pair-wise combinations of all genes were examined to determine the REO pattern of each gene pair within the sample. The within-sample REO of a gene pair (*i, j*) has only two possibilities, *G*_*i*_ > *G*_*j*_ or *G_*i*_* < *G*_*j*_, where *G*_*i*_ and *G*_*j*_ denote the expression values. If the number of RD samples with a certain REO pattern (*G*_*i*_ > *G*_*j*_ or *G*_*i*_ < *G*_*j*_) is significantly more than expected by chance, we define this gene pair as a stable gene pair of RD samples; stable gene pairs of pCR samples are defined in a similar manner. The significance of a REO in RD (or pCR) samples was determined using a binomial test (Bahn, 1969) as follows:

(1) P=1-∑i=0k-1(ni)⁢p0i⁢(1-p0)(n-i)

where *n* is the total number of samples with the RD (or pCR) status, *k* denotes the number of samples that have a certain REO pattern (*G_*i*_* > *G*_*j*_ or *G*_*i*_ < *G*_*j*_), and *p*_0_ denotes the probability of observing a gene pair with a certain REO pattern by chance (here, *p*_0_ = 0.5). Then the *P*-values were adjusted using the Benjamini and Hochberg (1995) procedure to control the false discovery rate (FDR).

We then defined stable-reversal gene pairs as pairs that had a significantly stable REO pattern in the pCR samples and RD samples, respectively, but had a reversal REO pattern between the two groups.

### Significant Majority Vote Rule

Based on the stable-reversal gene pairs between the pCR and RD, we developed an extremely resistant signature. A sample was identified as an extremely resistant sample, if the number of REOs of the signature gene pairs matching that of the extremely resistant group was significantly more than expected by chance. The threshold for identifying an extremely resistant sample was determined according to a binomial test as follows:

(2) P=1-∑i=0k-1(ni)⁢p0i⁢(1-p0)(n-i)

where *n* is the number of signature gene pairs and *k* is the number of gene pairs in the sample that match the REOs for the extremely resistant group. *p*_0_ (here, *p*_0_ = 0.5) is the probability of a gene pair having a certain REO pattern in a sample by chance.

### Survival Analysis

The distant relapse-free survival (DRFS), defined as the time from surgery to distant recurrence or the final documented date (censored), was used as a surrogate assessment of tumor response status (Liedtke et al., 2008). A log-rank test was used to assess the difference between the Kaplan–Meier estimates of DRFS in two different groups. The univariate Cox proportional-hazards regression model was used to calculate the hazard ratios (HRs) and their 95% confidence intervals (CIs).

## Results

### Development of the Resistant Signature

**FIGURE 1:**
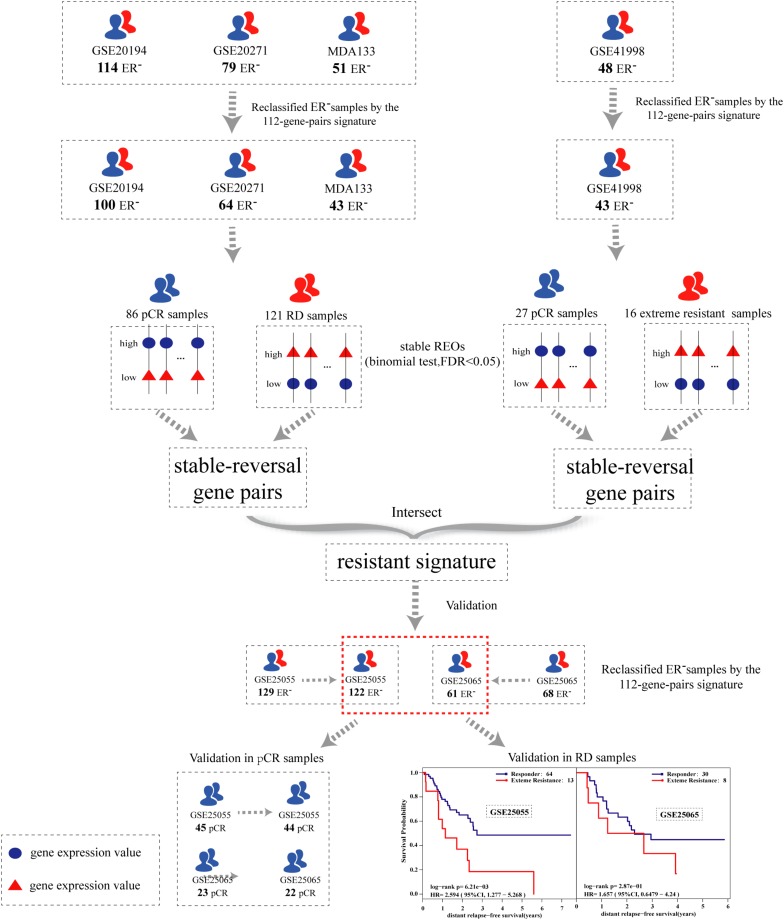
The flowchart for developing and validating the resistant signature.

The flowchart of the process used for developing and validating the resistance signature is shown in Figure 1. Using the 112-gene-pair signature for the ER status, 100, 64, 43, and 43 samples were re-determined as ER-negative samples from the GSE20194, GSE20271, MDA133, and GSE41998 dataset, respectively (Table 2).

**TABLE 2:** The ER-negative samples reclassified by the 112-gene-pairs signature from the IHC-determined ER-negative samples.

Usage	Dataset	Reclassified ER-negative sample size	pCR	RD
Training	GSE20194	100	43	57
	GSE20271	64	19	45
	MDA133	43	24	19
	GSE41998	43	27	16^a^
Validation	GSE25055	122	45	77
	GSE25065	61	23	38

*^a^PD and SD samples representing tumor residuals screened from RD samples.*

To identify an extremely resistant signature, we first extracted 169,222 gene pairs with stable (binomial test, FDR < 0.05) but reversed REOs between the pCR and RD group from the integrated data of the GSE20194, GSE20271, and MDA133 datasets, with 86 pCR samples and 121 RD samples in total. We then used the GSE41998 dataset to optimize the signature. Beside the pCR and RD category, the GSE41998 dataset also provided an evaluation of drug response criteria in solid tumors (RECIST), which divided the patients into four groups: complete response (CR), partial response (PR), stable disease (SD), and progressive disease (PD) (Watanabe et al., 2009). Among them, SD and PD indicated that the tumor area of the patients did not improve significantly but was increased after receiving NAC; therefore, we screened out the PD and SD samples from the RD samples as extremely resistant samples. We then extracted 30,588 stable-reversal gene pairs between the 16 extremely resistant samples and 27 pCR samples. Finally, 61 gene pairs that had consistent REO patterns between the above two lists of stable-reversal gene pairs were selected as the resistance signature, denoted as NR61. The details of NR61 are shown in Table 3. Each gene pair has a certain REO pattern in extremely resistant patients and a reversal REO pattern in responsive patients. Based on the significant majority vote rule (see section “Materials and Methods”), if more than 37 gene pairs (*P* < 0.05) of NR61 showed the same REO patterns as observed in extreme resistance, the sample was identified as extremely resistant; otherwise, it was considered a responder.

**TABLE 3:** Each pair of genes in NR61.

Gene 1	Gene 2	Gene 1	Gene 2	Gene 1	Gene 2
UBTD1	ACOX1	LAMA5	SMARCC1	LMAN2L	COBL
NOVA2	ADCY2	GPX5	SST	RBP3	PART1
TAS2R1	APLP1	GRIA1	SST	DNAH2	PART1
GCLM	ARL1	TMEM165	VAMP7	GCLM	CHIC2
RASL11B	CKB	STC1	VEGFB	ACKR4	TOX3
PTPRA	RCAN1	TGFB3	AKAP1	ATHL1	SLC43A3
AGPAT2	GNAQ	TPST2	SPOP	SLC30A1	ERGIC2
PLD2	GTF2F1	LETM1	SORBS2	FAM69A	CRNKL1
B4GALT5	HNRNPF	COPZ1	IQGAP1	VRK2	DPM3
NOS2	HSPA1L	GCLM	PRPF4B	SFXN3	CPVL
GCLM	IPO5	MICALL2	PRPF4B	TRAFD1	BSPRY
SYDE1	MAZ	IGSF3	ZNHIT3	GCLM	C5orf22
GTF2H3	NFIB	FAM69A	RNF14	SLC12A4	LMO3
MCAM	NFIB	TJP1	GCC2	SULT2B1	LMO3
TBC1D4	NFIB	LETM1	TOX4	SLC28A1	LMO3
NUAK1	NFIB	C10orf2	DCAF7	SEMA3F	FKBPL
FZD6	NUCB2	CPA3	SPAG5	GCLM	AIDA
CIAPIN1	PBX1	DUOX1	OR7E14P	P3H1	C17orf70
TRIT1	PBX3	MPPE1	KAT7	KREMEN2	IL17RC
GCLM	RBM3	GLTSCR1	XPO7	SMURF1	KLHL22
MMP16	RYR3				

*The expression patterns of these gene pairs is Gene 1 > Gene 2 in the extremely resistant samples and Gene 1 < Gene 2 in the response samples.*

Researchers (Tong et al., 2015) have proven that if two different regimens share one or several drugs, then the overlaps of the clinically relevant drug resistance genes (CRGs) for the two different regimens should be considered as the CRGs for the shared drug(s). We speculated that this is similar for clinically relevant drug resistance gene pairs (CRGPs). In this study, overlapping gene pairs between 169,222 CRGPs of PFAC and 30,588 CRGPs of PAC should thus be the CRGPs for PAC. Thus, the resistant signature that we developed is specific for predicting PAC resistance. However, the NR61 signature should be applicable for patients who received any combination of P, A, and C, as the extremely resistant patients identified by this signature showed multidrug resistant to P, A, and C.

### Performance of the NR61 Signature

In the GSE25055 and GSE25065 datasets, 122 and 61 ER-negative breast cancer samples were separately re-determined using the 112-gene-pair signature (Table 2) and were used to validate NR61. Among these re-determined ER-negative samples, the NR61 signature could correctly classify 44 (97.8%) out of 45 pCR samples and 22 (95.7%) out of 23 pCR samples as responder samples, which showed that NR61 can effectively identify patients that completely responded to PAC NAC.

**FIGURE 2:**
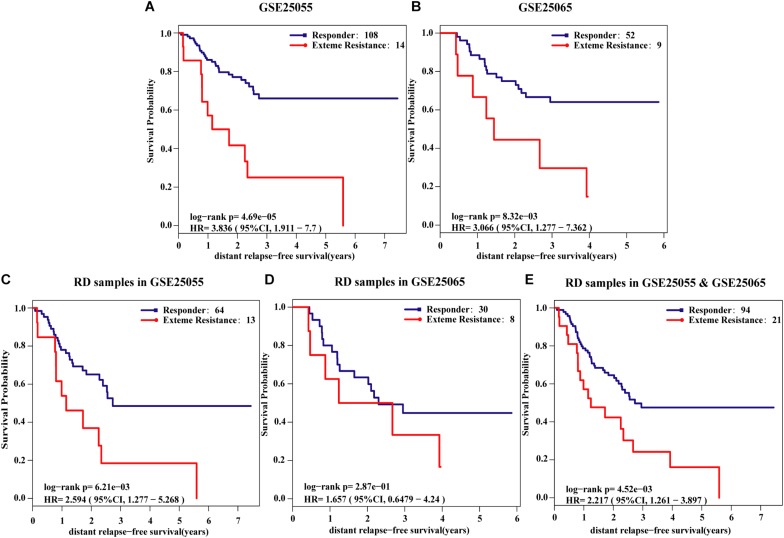
Kaplan–Meier estimates of distant relapse-free survival (DRFS). DRFS curves for responder and extreme resistance in **(A)** GSE25055; **(B)** GSE25065; **(C)** RD samples of GSE25055; **(D)** RD samples of GSE25065; **(E)** integrated RD samples of GSE25055 and GSE25065.

The survival analysis was then used to validate the NR61 signature, assuming that the responsive patients have a better prognosis than the extremely resistant patients. First, the survival analysis was performed in all re-determined ER-negative breast cancer patients. In the GSE25055 dataset with 122 ER-negative breast cancer patients, 108 and 14 patients were classified as responders and extremely resistant, respectively. The extremely resistant patients had a significantly shorter DRFS time than the responders (log-rank *P* < 0.01; HR = 3.84; 95% CI = 1.91–7.70; Figure 2A). Similar results were obtained in the GSE25065 dataset with 61 ER-negative breast cancer patients (log-rank *P* < 0.01; HR = 3.07; 95% CI = 1.28–7.36; Figure 2B).

To avoid the impact of pCR patients and further demonstrate the poor prognosis of extremely resistant patients, the survival analysis was limited to RD patients. For the 77 RD patients with ER-negative breast cancer in the GSE25055 dataset, 64 and 13 patients were classified into the responder and extremely resistant group, respectively. Survival analysis showed that the DRFS time of the extremely resistant group was significantly shorter than that of the responders (log-rank *P* < 0.01; HR = 2.59; 95% CI = 1.28–5.27; Figure 2C). In the RD samples from the GSE25065 dataset with 38 ER-negative breast cancer patients, the NR61 signature stratified 30 and 8 RD patients into the responder and extremely resistant groups, respectively. Survival analysis showed that, in this dataset with a small sample size (low statistical power) there was a trend of difference in the DRFS time between the responder and extremely resistant groups (log-rank *P* = 0.29; HR = 1.66; 95% CI = 0.65–4.24; Figure 2D). In the integrated data of the two datasets with 94 responders and 21 extremely resistant patients in total, identified from the RD patients, the former had significantly longer DRFS than the latter (log-rank *P* < 0.01; HR = 2.22; 95% CI = 1.26–3.90; Figure 2E). This result indicates that NR61 well divided the RD samples into two categories, one of which is the PR to NAC with a good prognosis, whereas the other has a very poor prognosis, which is extreme resistance.

In the validation dataset where a number of patients received PA rather than PAC, the extremely resistant patients who were multidrug resistant to P, A, and C should have a poor prognosis, while those patients who were resistant to P and A but sensitive to C would be classified into the response group. The patients under a treatment of PA should have a poor prognosis. However, we still observed the extremely resistant group had a significantly longer survival than the responder group, even though the latter included some patients with poor prognosis.

All the above results indicate that the extremely resistant patients identified by NR61 cannot benefit from the PAC NAC treatment. The NR61 signature is thus expected to assist physicians in choosing treatment plans for ER-negative breast cancer patients in clinical practice. If a patient is judged as extremely resistant by NR61, accepting PAC NAC may only cause complications and a loss of the best time for surgery. For these patients, other chemotherapeutic regimens or direct surgery might be more sensible options.

### Correlation of NR61 With HER2 Status and PAM50 Subtype

As HER2 status is an important prognostic and predictive signature, we evaluated the performance of NR61 in HER2− and HER2+ patients, respectively. We found that all 61 ER-negative breast cancer samples of GSE25065 were HER2− and that 115 of 122 ER-negative breast cancer samples in GSE25055 were HER2−. In the 115 HER2+ patients, the survival of the responder group and the extremely resistant group as identified by NR61 was significantly different (Supplementary Figure 1A). A similar result was found in 74 RD samples (Supplementary Figure 1B). For another seven patients in GSE25055, the HER2 status of three patients was positive, and four patients were uncertain. All of these seven patients were classified into the responder group by NR61.

In addition, we counted the number of samples for each PAM50 subtype in the responder group and in the extremely resistant group as reclassified by NR61. In the responder group of the GSE25055 dataset, the sample sizes of Normal, Luminal A, Luminal B, HER2, and basal-like were 1, 0, 0, 1, and 12, respectively. In the extremely resistant group, the sample size corresponding to these subtypes was 8, 0, 0, 8, and 92, respectively. A Chi-square test showed no statistically significant difference in the sample distribution of each PAM50 subtype between the responder group and the extremely resistant group (*P* = 0.9986, Supplementary Figure 2A). Similar results were also observed in the GSE25065 dataset (*P* = 0.1213, Supplementary Figure 2B). This result indicates that there is no relationship between NR61 and PAM50 subtypes.

## Discussion

In this study, we developed a qualitative drug resistant signature (NR61), which could well predict the ER-negative breast cancer patients who were extremely resistant to PAC NAC. Based on this signature a total of 183 ER-negative patients in the two validation datasets could be divided into responder and extremely resistant patients. Our research showed that the DRFS time of the extremely resistant group was significantly shorter than that of the responders. Patients identified with extreme resistance should be recommended other treatment schemes to avoid unnecessary suffering and expenses. Additionally, this signature can correctly identify almost all patients who can completely respond to PAC NAC.

Our qualitative transcriptional signature based on the within-sample REOs is robust against batch effects (Chen et al., 2017; Cheng et al., 2017; Guan et al., 2018) and could be performed for the individual analysis of ER-negative breast cancer, which is of great value for clinical application. The REO-based signatures may lose some so-called “subtle” quantitative information of gene expression measurements. However, the “subtle” quantitative information is often unreliable because it is affected by the high variations in measurement and batch effects, the proportions of tumor epithelial cells in clinical tissue samples, partial RNA degradation during specimen preparation and storage, and the amplification bias of low-input RNA (Freidin et al., 2012; Chen et al., 2017). Even the ratios of the expression values of gene pairs are affected by batch effects (Loven et al., 2012; Qi et al., 2016). Thus, this apparent disadvantage of REO analysis is actually a unique advantage in terms of robustness (Chen et al., 2017).

In this study, PD and SD samples screened from RD samples were defined as extremely resistant to PAC NAC. The pCR-RD system is based on microscopic observation and a large number of patients are diagnosed with RD. However, in the image-based RECIST system (Watanabe et al., 2009), PD is defined as at least a 20% increase in the sum of the diameters of target lesions after receiving NAC, and SD is defined as neither sufficient shrinkage to qualify for PR (at least a 30% decrease in the sum of diameters of target lesions) nor sufficient increase to qualify for PD, both of which are less sensitive to NAC. Therefore, it is reasonable to screen PD and SD patients from RD patients as extremely resistant, as used in this study. However, there is only one dataset with information of both RECIST and pCR-RD. Thus, we used the DRFS to evaluate whether the identified patients can benefit from PAC NAC.

Due to the lack of RNA-seq data with suitable drug response information, we only tested the NR61 signature in the microarray data measured on the Affymetrix platform. In future, we will collect the breast cancer expression data from the RNA-seq platform to optimize our signature, in order to improve its cross-platform ability.

## Conclusion

In summary, the NR61 signature could be used to robustly identify patients who are extremely resistant to PAC NAC among ER-negative breast cancer patients. These patients are highly unlikely to benefit from the PAC NAC regimen and should thus be recommended other therapeutic regimens. The clinical value of the NR61 signature for extreme resistance to the PAC NAC regimen thus deserves further validation.

## Data Availability Statement

The datasets analyzed in this study could be found in the Gene Expression Omnibus (GSE20194, GSE20271, GSE41998, GSE25055, and GSE25065) and MD Anderson Cancer Center (https://bioinformatics.mdanderson.org/public-datasets/).

## Author Contributions

ZG and YC conceived the project. YC and HC performed the computational experiments. YC and JL designed the data analyses. QG and JH interpreted the data. YC, HC, and ZG wrote the manuscript. All authors contributed to the preparation of the manuscript, and read and approved the final manuscript.

## Conflict of Interest

The authors declare that the research was conducted in the absence of any commercial or financial relationships that could be construed as a potential conflict of interest.

## Footnotes

1 http://www.ncbi.nlm.nih.gov/geo/

2 https://bioinformatics.mdanderson.org/pubdata.html
