# Supplementary material for: A Qualitative Transcriptional Signature for Predicting Extreme Resistance of ER-Negative Breast Cancer to Paclitaxel, Doxorubicin, and Cyclophosphamide Neoadjuvant Chemotherapy
Source: Front Mol Biosci. 2020 Mar 25;7:34. doi: 10.3389/fmolb.2020.00034 (PMC7109260; doi:10.3389/fmolb.2020.00034)
Supplement: Supplementary file 1 [file Data_Sheet_1.PDF]

Supplementary Material

Supplementary Figures

Supplementary Figure S1 Kaplan-Meier estimates of distant relapse - free survival (DRFS)

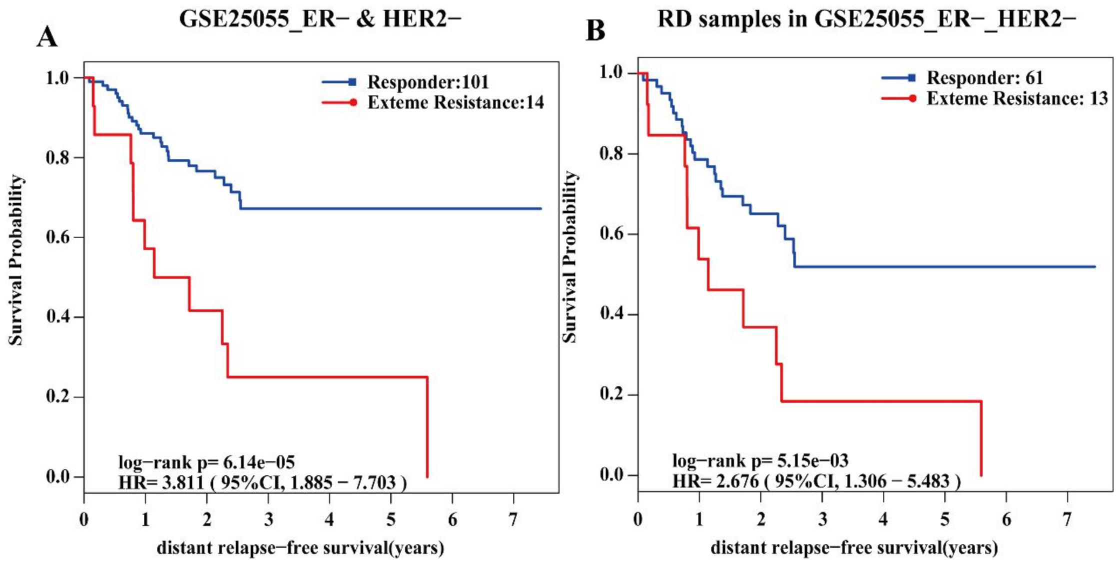

Supplementary Figure S1. DRFS curves for responder and extreme resistance in (A)ER- and HER2- patients in the GSE25055 dataset; (B)RD samples of ER- and HER2- patients in the GSE25055 dataset.

Supplementary Figure S2. The samples distribution of each PAM50 subtype in response group and extremely resistant group

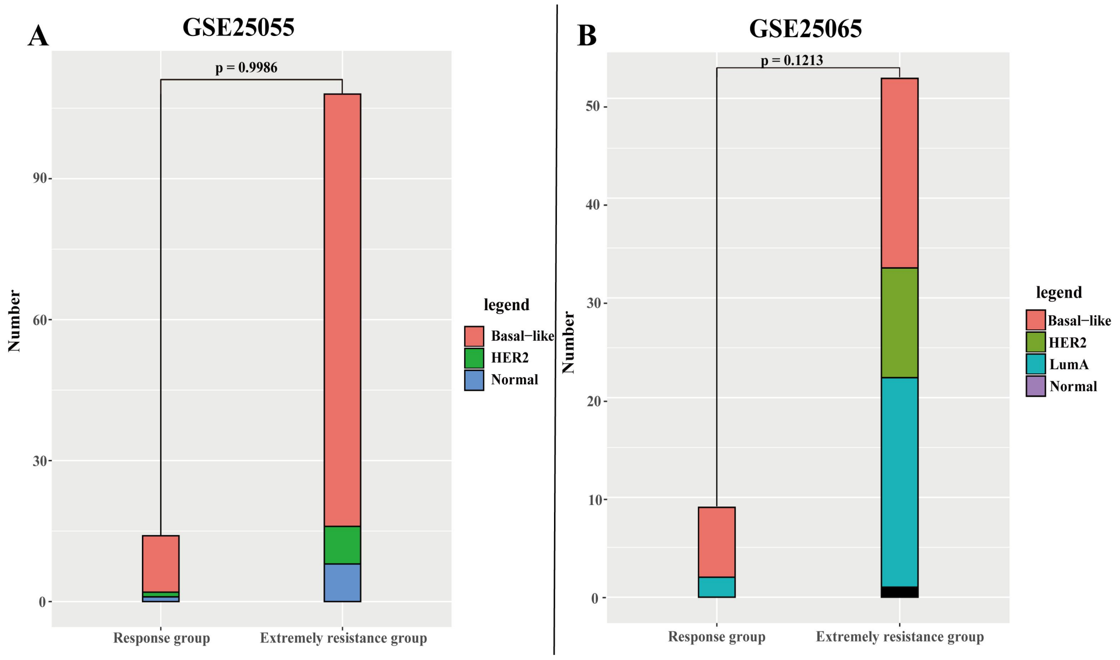

Supplementary Figure S2. Chi-square test of the samples distribution of each PAM50 subtype between response group and extremely resistant group in (A) GSE25055; (B) GSE25065.
